# Loss of the RNA Binding Protein HuR in Early Murine Limb Mesenchyme Does Not Affect Development but Leads to Impaired Bone Homeostasis in Adulthood

**DOI:** 10.1096/fj.202500780RR

**Published:** 2025-11-20

**Authors:** Shijian Fu, Kirsty A. Johnson, Phaedra Winstanley‐Zarach, Ufuk Ersoy, Elena Adlmanninger, Benjamin T. McDermott, Craig Keenan, Aphrodite Vasilaki, Ioannis Kanakis, Peter I. Milner, Rob J. van’t Hof, Dimitris Kontoyiannis, George Bou‐Gharios, David A. Turner, Simon R. Tew

**Affiliations:** ^1^ Department of Musculoskeletal and Ageing Sciences, Institute of Life Course and Medical Sciences University of Liverpool Liverpool UK; ^2^ Medical School Edge Hill University Ormskirk UK; ^3^ Chester Medical School, Faculty of Health, Medicine and Society University of Chester Chester UK; ^4^ Vanthof Scientific Torun Poland; ^5^ Institute for Fundamental Biomedical Research (IFBR) Biomedical Sciences Research Centre “Alexander Fleming” Vari Greece; ^6^ Department of Genetics, Development & Molecular Biology, School of Biology Aristotle University of Thessaloniki Thessaloniki Greece

## Abstract

In this study, we examined how a critical posttranscriptional regulator, the RNA‐binding protein HuR (gene name Elavl1), contributes to the development and maintenance of limb skeletal tissue. Using the Prx1‐Cre knockout model, we examined the effect of germline knockout (Elavl1KO) and limb mesenchyme‐specific knockout (MSC‐Elavl1KO) of HuR on limb development. We found that Elavl1KO disrupted the development of the limb skeleton and was associated with a loss of signaling from the apical ectodermal ridge (AER). In contrast, MSC‐Elavl1KO did not appear to affect skeletal development. Mature MSC‐Elavl1KO mice appeared healthy, but their limb skeleton exhibited abnormal bone structure in both males and females at 2.5 months of age. Osteoblasts isolated from MSC‐Elavl1KO mice exhibited lower expression of osteoblastic marker genes, and their ability to generate a mineralized matrix was markedly impaired. RNA‐Seq analysis of these osteoblasts demonstrated that loss of HuR substantially influenced their transcriptome, affecting genes associated with a wide range of cellular processes. Finally, using siRNA knockdown in the human MG63 cell line, we identified that loss of HuR leads to increased mRNA turnover of the osteoblastic transcription factor Runx2. Overall, the study has demonstrated a critical role for HuR‐mediated posttranscriptional control in skeletal development and homeostasis, but finds that its expression in mesenchyme‐derived cells only becomes critical in mature skeletal tissue.

## Graphical abstract

The RNA‐binding protein HuR (Elavl1) is essential for limb development. In germline HuR knockouts, apical ectodermal ridge signaling is lost, though mesenchymal signals persist. Mesenchyme‐specific HuR deletion does not affect embryonic limbs, but adult mutants are affected and exhibit markedly reduced trabecular bone density. These findings point toward a critical role for HuR expression in nonmesenchymal tissues during limb development, but its presence in mesenchymal‐derived cells is important for the healthy homeostasis of the mature limb skeleton. Created using BioRender.

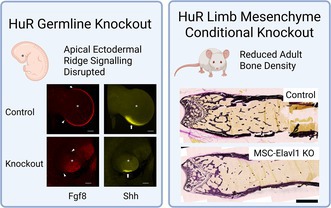

## 1 Introduction

The development and maintenance of the musculoskeletal system involve intricately controlled processes, characterized by precise growth and patterning of embryonic tissues and tightly regulated remodeling processes in adulthood. These processes are driven and regulated by a wide range of signals that combine to ensure functional modulation of transcripts and proteins in the different cell types that produce and maintain the tissues. One important tier of regulation is the posttranscriptional control of mRNA turnover and translation. Posttranscriptional control, therefore, shapes the steady state levels, response kinetics, and localization of transcripts and proteins [1].

Regulation of posttranscriptional gene regulation, through the actions of noncoding RNAs, has been identified as being important in musculoskeletal tissue development, growth, and pathology [2, 3, 4, 5, 6, 7]. Many RNA‐binding proteins (RNABPs) are also able to interact with mRNAs and influence their turnover and translation. Mouse knockout models exist for several posttranscriptional regulatory proteins, and in some cases, no obvious musculoskeletal phenotype is observed, suggesting that there is either no role or that they have redundant effects in these tissues [8]. Loss of some RNABPs can lead to musculoskeletal disruption indirectly, typically due to perturbed regulation of inflammatory factors. For instance, knockout of tristetraprolin (ZFP36) leads to elevated systemic tumor necrosis factor α (TNFα) protein levels due to impaired turnover of the TNFα mRNA, resulting in an inflammatory syndrome that includes aggressive synovial joint destruction [9]. Another RNABP, KHSRP has also been linked to inflammatory arthritis and systemic lupus [10, 11]. A direct effect of the tristetraprolin family member ZFP36L1in joint tissues themselves has been identified, where it has been shown to contribute to the development of osteoarthritis in mice by regulating mRNA encoding the chaperone protein HSPA1A1A [12]. One notable example of evidence for an RNABP controlling skeletal development is found in the ubiquitously expressed protein HuR (ELAVL1). Knockout of HuR in the epiblast leads to a disruption of skeletal development, along with effects in several other tissues [13]. Loss of HuR leads to impaired growth and mineralization of skeletal structures in the limbs, ribs, spine, and skull. Syndactyly is also observed, indicating a role for HuR‐mediated mRNA regulation in the control of tissue patterning [13]. In vivo analysis of HuR knockout in skeletal muscle did not identify substantial changes in muscle size or appearance, but did result in an increase in the proportion of type I muscle fibers found in the soleus, leading to the mice having a greater anaerobic capacity compared to wild‐type controls [14].

Despite the evidence for HuR's role in controlling musculoskeletal tissue development, our understanding of the mechanisms underlying this is incomplete. Limb development begins following outgrowths of mesoderm from the flanks of the embryo in structures known as limb buds. Musculoskeletal tissues derive from condensations of mesenchyme within these structures [15]. Several mechanisms have been identified that control outgrowth and patterning of the limb skeleton, which involves signals deriving from within the mesenchyme as well as from the ectoderm at the tip of the growing limb bud. We were interested in the role HuR plays in these mesenchyme‐derived musculoskeletal tissues when knockout during development. We have therefore used Cre recombinase‐mediated knockout of HuR in developing limb mesenchyme using a well‐established Prx1‐Cre model, which can lead to conditional gene knockout in the early limb mesenchyme [16]. We found to our surprise that specific loss of HuR in developing skeletal structures did not replicate the negative effects on limb development previously observed in the epiblast knockout [13]. However, analysis of adult mice demonstrated a dramatic loss of trabecular bone density in knockout animals, indicating that an important functional role for HuR in controlling bone homeostasis exists, which is mediated through cells of mesenchymal origin.

## 2 Materials and Methods

### 2.1 Mice

Mixed background C57BL/6 X CBA B6BAF1 were used for all murine transgenic studies, and all animal procedures were licensed by the UK Home Office under the Animal (Scientific Procedures) Act 1986; project license numbers (PPL) 70/7288 and 70/9047. Mice were housed at a specific pathogen‐free (SPF) mouse facility in the Biomedical Services Unit (BSU) at the University of Liverpool. Mice received 12 h of light and dark and 45% ± 10% humidity at a temperature of 22°C ± 2°C. Access to water and food was ad libitum.

Cryopreserved HuR^fl/WT^ embryos [13] were re‐derived at the University of Liverpool. Mixed background females were housed with vasectomized males and pseudo‐pregnant females identified by the presence of a copulation plug. Embryos were transferred into pseudo‐pregnant females, and resulting pups were backcrossed to generate HuR^fl/fl^ animals.

6‐week‐old, male Prx1‐Cre mice [16] were purchased from The Jackson Laboratory (Jax stock #005584). Male Prx1‐Cre were bred with HuR^fl/fl^ females to generate HuR^fl/WT^Prx1‐Cre^+/−^ F1 mice. Initially, these F1 progeny were backcrossed to HuR^fl/fl^ mice to produce 25% of embryos with HuR^fl/f^Prx1‐Cre^+/−^ that would have recombination at the flox sites. The effects of the Cre allele being inherited both maternally and paternally were investigated by analysis of embryos, as maternal inheritance can result in germline transmission of the Cre allele [16]. Adult HuR^fl/fl^Prx1‐Cre^+/−^ were also studied and used for breeding to increase HuR recombination frequency. All mice were genotyped using DNA isolated from ear notches and polymerase chain reaction for Cre and HuR^fl/fl^. Embryos were genotyped using DNA from the yolk sac or tail tissue, depending on developmental stage.

### 2.2 Whole Mount Staining

Embryos were stained with alcian blue (whole mount), adapted from a previously described protocol [17]. Briefly, embryos were fixed in 95% ethanol, placed in 100% acetone for 2 days, then stained for 3 days at 37°C in freshly prepared staining solution (1 volume 0.3% alcian blue 8GX in 70% ethanol, 1 volume 0.1% alizarin red S in 95% ethanol, 1 volume glacial acetic acid, and 17 volumes 70% ethanol). Embryos were then washed with distilled water and cleared in a 1% solution of potassium hydroxide for 12–48 h, then processed through 20%, 50% and 80% glycerol/1% potassium hydroxide solution for around 4 days prior to storage in 100% glycerol.

### 2.3 Embryo Whole Mount mRNA In Situ Hybridization

Female HuR^fl/fl^ Prx1‐Cre^+/−^ mice were mated to HuR^fl/fl^ Prx1‐Cre^−/−^ males, and embryos were collected at E9.5—E11.5. The embryos were fixed in 4% formaldehyde (28 908, ThermoFisher Scientific) at 4°C overnight, then dehydrated with serial methanol and stored in 100% methanol (34860‐1 L‐R, Sigma) at −20°C. Embryos were rehydrated and incubated in 10ug/ml proteinase K solution (1 864 261, Invitrogen) and postfixed with 4% formaldehyde for 20 min. In situ HCR v3.0 was performed using the protocol of Choi and colleagues [18] adapted to use an increased probe concentration during hybridization. Briefly, embryos were hybridized with 10 nM probe mixture overnight. After that, excessive probes were washed off using the probe wash buffer as per the original protocol. Signals were subsequently amplified by applying a fluorescently labeled hairpin amplifier overnight at room temperature. The embryos' cell nuclei were then stained with DAPI (ab228549, Abcam) before imaging. HCR probe sets were designed following previously reported protocols [18, 19]. All oligonucleotide probe set sequences and hairpin information are provided in Table S1. Probes were purchased from Sigma, and other related reagents were obtained from Molecular Instruments, CA.

### 2.4 Immunofluorescence

Embryos were fixed in 4% formaldehyde for 1 h at 4°C, washed, and stored in 0.0025% Triton X‐100 in PBS (PBS‐Tr) at 4° *C.* Prior to immunofluorescence staining, embryos were permeabilized with 0.25% Triton X‐100 in PBS for 30 min and then blocked with 2% bovine serum albumin in PBS solution for 1 h before incubation with 1:100 Fgf8 antibody (MAB323, R&D Systems, UK) overnight at 4°C. The embryos were then washed four times with PBS‐Tr for 1 h each, and then incubated with fluorescently labeled donkey anti‐mouse antibody (A‐21202, Invitrogen, UK) and DAPI overnight at 4°C. The following day, the embryos were washed four times with PBS‐Tr, each wash lasting 1 h prior to imaging.

### 2.5 Confocal Microscopy

Post HCR or immunofluorescence, the embryo's limb buds were imaged on an Andor Dragonfly spinning disc confocal microscope (Andor, Oxford Instruments) mounted on an inverted Leica DMi8 base using a 10× (0.45NA) objective. Alexa Fluor−405, −594, and −647 were excited with 405, 561, and 637 nm laser diodes, respectively, and emitted light reflected through 450/50, 600/50, and 700/25 nm bandpass filters, respectively, using a pinhole set to 40 nm. An iXon 888 EM‐CCD camera was used to collect emitted light, and data were captured using Fusion version 5.5 (Bitplane) with subsequent image analysis performed using Imaris viewer (version 10.1.0).

### 2.6 Histology and Immunohistochemical Analysis

Embryos collected for histological analysis were fixed in 10% neutral‐buffered formalin for 48 h and stored in 70% ethanol. Samples were processed into paraffin wax and sectioned at 20 μm thickness. Embryo sections were stained with safranin‐O/fast green or Toluidine Blue. Sections from E13.5 and E16.5 embryos were stained with a monoclonal antibody to HuR (sc‐5261, Santa Cruz Biotechnology, Dallas, TX), used at a 1:100 dilution using a mouse‐on‐mouse immunohistochemical staining kit, following the manufacturer's instructions (BMK‐2202, Vector Labs, Newark, CA).

### 2.7 Measurement of Bone Formation in Young Adult Mice

Adult mice's isolated hind limbs were fixed as described above, but prior to histological analysis, the specimens were decalcified in 10% EDTA for 3 weeks. Samples were then processed into paraffin wax, and sections were stained with safranin‐O and fast green, toluidine blue, or processed for immunohistochemistry as described below. Alternatively, mineralized limb tissues were processed into methylmethacrylate resin and sectioned as previously described [20] and stained with Von Kossa reagent to identify mineralized bone matrix. All histologically stained slides were scanned on an Axio Scan Z1 slide scanner (Zeiss, UK). Knee joint integrity scoring was performed according to OARSI guidelines [21]. Sections from adult limbs were examined by immunohistochemistry to detect HuR protein as described above and Runx2 (ab192256, Abcam, Cambridge, UK), which was visualized using a standard Vectorstain ABC approach (PK‐6100, Vector Labs). Immunohistochemistry photomicrographs were imaged with a Zeiss Axiocam 105 microscope (Carl Zeiss Microscopy Ltd., Cambridge, UK). Pixel classifiers were trained within QuPath software and used for semi‐quantification of positive staining in different areas of each sample.

### 2.8 Microcomputed Tomography (μCT)

Prior to decalcification, the hind limbs were scanned in 70% ethanol using either a SkyScan 1272 high‐resolution micro‐computed tomography (μCT) scanner (SkyScan, Aartselaar, Belgium) or a NeoScan N 80 (Neoscan, Mechelen, Belgium). Samples from female mice were scanned using the SkyScan 1272 at a voxel size of 4.5 μm. Scans were acquired at 50 kV and 200 μA, with a 0.5 mm aluminum filter and a rotation step of 0.3° over a 180° rotation. Owing to COVID‐19 restrictions, a full series of male samples could not be collected until a later time point. For technical reasons, this meant that imaging of the male mice was performed using the NeoScan 80 as follows. To measure limb length, male hind limbs were scanned using the NeoScan 80 at a voxel size of 12 μm, with acquisition parameters set at 67 kV and 200 μA, using a 0.5 mm aluminum filter, 2 × 2 binning, and a rotation step of 0.4° over a 180° rotation. For male bone morphology analysis, the distal femur was scanned at a higher resolution with a voxel size of 5 μm, maintaining the same acquisition settings. Three‐dimensional images were reconstructed from μCT data using 3D Slicer software. Quantification of bone architecture was conducted as previously described [22]. However, as we observed a reduction in bone length of 6% at 2.5 months of age, and 7% at 6 months of age, the size of the volume of interest (VOI) used for the analysis of bone morphometry was reduced from 200 slices to 188 and 186 slices, respectively. In addition, the distance of the VOI to the growth plate was reduced from 180 to 170 and 167 μm, respectively.

### 2.9 Cell Culture

Primary murine osteoblasts were isolated from minced bone chips collected from 2.5‐month‐old animals. Cells were allowed to grow out from the tissue and then replated 1 × 10^5^ cells in 12‐well plate for experiments. Primary osteoblasts were induced to produce a mineralized extracellular matrix by culturing in α Minimum Essential Medium supplemented with 10% fetal bovine serum, 10 units/mL penicillin, 0.1 mg/mL streptomycin, 5 mM beta glycerophosphate, and 50 ug/ml ascorbate 2‐phosphate for 3 weeks. Mineralization of the osteoblasts' matrix was detected by staining of cell layers with Alizarin Red S solution. MG63 osteosarcoma cells were maintained in standard growth media prior to use in siRNA knockdown experiments. Using cells cultured in 12‐well plates, control or HuR‐specific siRNAs were transfected (22.5 pmol/well) into the MG63 cells using 2 μg/mL DharmaFECT siRNA transfection reagent (Horizon Discovery, Cambridge, UK), and the cells were cultured for 72 h before being incubated with 10 uM actinomycin D for up to 24 h.

### 2.10 qRT‐PCR

Total RNA was isolated from cell layers using TRIzol (ThermoFisher, UK). cDNA was synthesized using MMLV Reverse transcriptase, primed with random hexamer oligonucleotides. Quantitative real‐time PCR analysis of the cDNA was performed in Takyon ROX SYBR 2X MasterMix dTTP blue (Eurogentec, UF‐RSMT‐B0701) with gene‐specific primers obtained from Eurogentec (Seraing, Belgium) using a Roche Lightcycler 96 thermal cycler. Relative quantification of transcripts was determined using the 2^−ΔCt^ method [23]. Primer sequences were as followed. Mouse Elavl1 5′‐ATGAAGACCACATGGCGGAAGACT‐3′ and 5′‐AGTTCACAAAACCGTAGCCCAAGC‐3′, mouse ALP 5′‐ACACCTTGACTGTGGTTACTGCTGA‐3′ and 5′‐CCTTGTAGCCAGGCCCGTTA‐3′, mouse RUNX2 5′‐GACGAGGCAAGAGTTTCACC‐3′ and 5′‐GGACCGTCCACTGTCACTTT‐3′, mouse Collagen1α1 5′‐TTCTCCTGGCAAAGACGCACTCAA‐3′ and 5′‐AGGAAGCTGAAGTCATAACCGCCA‐3′, mouse osteocalcin 5′‐CTCTGTCTCTCTGACCTCACA‐3′ and 5′‐CAGGTCCTAAATAGTGATACC‐3′, mouse osterix 5‐GAAAGGAGGCACAAAGAAG‐3′ and 5′‐CACCAAGGAGTAGGTGTGTT‐3′, mouse RPL32 5′‐TTCTTCCTCGGCGCTGCCTACGA‐3′ and 5′‐AACCTTCTCCGCACCCTGTTGTCA‐3′, human Elavl1 5′‐TTCACCACCAGGCGCAGAGA‐3′ and 5′‐GAGCCCGCTCATGTGATCGA‐3′, human osteocalcin 5′‐CAAAGGTGCAGCCTTTGTGTC‐3′ and 5′‐TCACAGTCCGGATTGAGCTCA‐3′, human ALP 5′‐ACCATTCCCACGTCTTCACATTT‐3′ and 5′‐AGACATTCTCTCGTTCACCGCC‐3′, human RUNX2 5′‐GGAGTGGACGAGGCAAGAGTTT‐3′ and 5′‐AGCTTCTGTCTGTGCCTTCTGG‐3′, human Collagen1α1 5′‐GTTCAGCTTTGTGGACCTCCG‐3′ and 5′‐GATTGGTGGGATGTCTTCGTCT‐3′, human COX7C 5′‐CAACCTCTGTGGTCCGTAGG‐3′ and 5′‐ACTGAAAATGGCAAATTCTTCCCAG‐3′.

### 2.11 Western Blotting

Proteins were isolated from cell layers either as part of the TRIzol process or through direct cell layer extraction in SDS sample buffer. Proteins were separated on 4%–12% Bis/Tris gradient gels (Thermo Fisher, UK) and then blotted onto a nitrocellulose membrane. Blots were blocked using Intercept blocking Buffer (927–60 001, Licor) and then probed with primary antibodies HuR (ab200342, Abcam or sc‐5261, Santa Cruz, 1:500), RUNX2 (ab192256, Abcam, 1:500), Collagen I (ab34710, Abcam, 1:500), αTubulin (ab4074, Abcam or T6074, Sigma, 1:1000), followed by fluorescently conjugated secondary antibodies (926–32 210 and 926–68 071, Licor, 1:10000). Fluorescence signal was imaged using a Li‐Cor CLX instrument (Li‐Cor Biotechnology, Cambridge UK).

### 2.12 Transcriptomic Analysis

Total RNA from primary murine osteoblasts was used to generate dual‐indexed, strand‐specific RNA‐Seq libraries using NEBnext polyA selection and Ultra II directional RNA library preparation kits (New England Biolabs). Libraries were sequenced on an Illumina NovaSeq instrument using SP chemistry. FastQ files containing trimmed reads were prepared and then aligned using StarAligner v.2.7.3a. Reads were aligned to the murine Mus_musculus_GRCm39_dna_sm_primary_assembly genome. Differential transcript analysis was then performed using DeSeq2, and gene ontology enrichment analysis was performed using ClusterProfiler (version 4.12.6) and REVIGO (version 1.8.1) [24] packages within R version 4.4.0. Read data contained in trimmed FastQ files have been deposited on ArrayExpress with the accession number E‐MTAB‐14888 (https://www.ebi.ac.uk/biostudies/arrayexpress/studies/E‐MTAB‐14888).

## 3 Results

### 3.1 The Knockout of HuR in the Germline Through Maternal Transmission of the Prx‐1Cre Allele Leads to Severely Disrupted Skeletal Development

**FIGURE 1:**
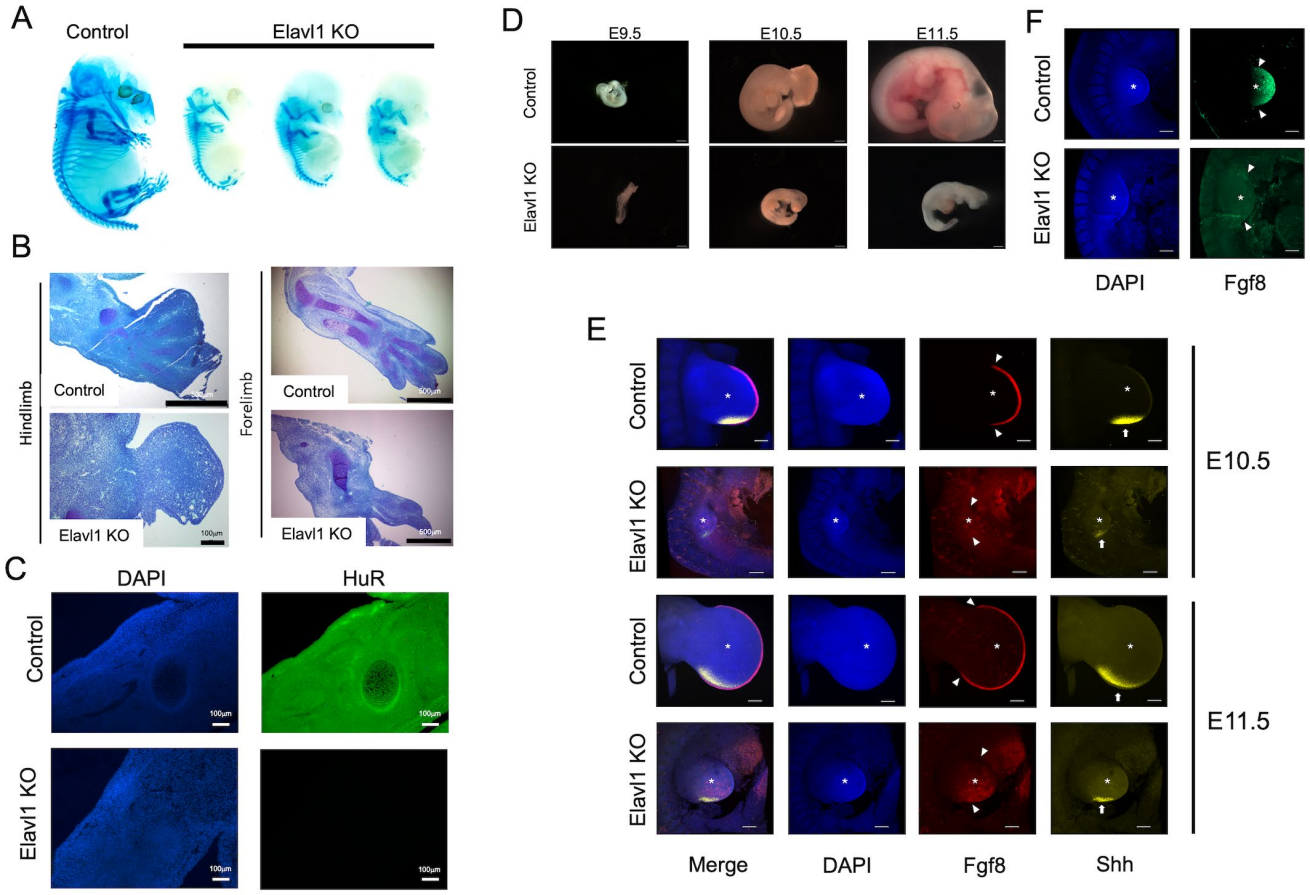
Effect of germline Cre recombination in HuR^fl/fl^ mice (Elavl1 KO) through maternal inheritance of Prx1‐Cre. (A) Whole‐mount embryos at E13.5 stained with alcian blue. (B) Toluidine blue‐stained sections of E13.5 embryo forelimbs and hindlimbs. (C) Immunofluorescence detection of HuR in limb sections from E13.5 embryos. (D) Brightfield micrographs demonstrating morphology changes in Control and Elavl1 KO embryos isolated from E9.5 to E11.5. Scale bar = 500 μm. (E) Whole‐mount in situ hybridization chain reaction detection of limb bud markers mRNA in E10.5 and E11.5 embryos. Asterisk: Limb bud, Triangle: Apical ectodermal ridge, Arrowhead: The zone of polarizing activity. Scale bar = 150 μm. (F) Whole‐mount immunofluorescence detection of Fgf8 protein in E10.5 embryos. Asterisk: Limb bud, Triangle: Apical ectodermal ridge. Scale bar = 150 μm.

To test whether conditional knockout of HuR in the mesoderm of the limb bud would replicate the limb phenotype observed previously in conditional knockouts of HuR in the epiblast, we generated mouse embryos where the Prx1‐Cre was inherited maternally on HuR^fl/fl^ background (subsequently referred to as Elavl1KO), which has been demonstrated to result in germline transmission [16]. Of four E13.5 litters where Cre was inherited maternally, three litters contained embryos with notable growth retardation (6 of a total of 24 embryos, Figure 1A), consistent with the results of previous knockouts of HuR at very early skeletal developmental [13]. Histological examination of the limbs demonstrated pronounced disruption to skeletal structures in the Cre‐positive embryos (Figure 1B). Immunofluorescent localization of HuR in E13.5 limbs confirmed widespread loss of expression in the Elavl1KO embryos (Figure 1C).

We next examined how limb developmental marker expression was affected in the Elavl1KO embryos at the early stages of limb bud formation. We initially attempted to analyze E9.5 Cre‐positive embryos, but the extent of their developmental retardation led to challenges in determining limb bud structures. We instead examined knockout embryos at E10.5 and E11.5, and were able to visualize the limb buds, although the knockout embryos were smaller than the wild types (Figure 1D). We examined the distribution of Fgf8 mRNA, a marker of the apical ectodermal ridge (AER), using in situ hybridization chain reaction (HCR). At both stages, we found a characteristic crescent distribution across the distal tip of the limb buds of Control embryos, but could not detect Fgf8 expression in the knockout embryo limb buds (Figure 1E). We also assessed the levels of Shh mRNA, a marker of the zone of polarizing activity (ZPA) in the posterior part of the limb bud mesenchyme, using HCR. Although knockout limb buds were smaller at each stage, we found Shh expression in both Control and knockout embryos (Figure 1E), indicating that the ZPA still formed in the absence of HuR. To further confirm the effect of HuR on AER signaling, we performed whole‐mount immunofluorescence analysis of Fgf8 protein in E10.5 embryos. Consistent with our findings at the mRNA level, we found that Fgf8 protein was present at the distal tip of Control mouse limb buds but could not be detected in those from knockout animals (Figure 1F).

### 3.2 Limb Mesenchyme‐Specific Knockout of HuR Through Paternal Transmission of Prx1‐Cre Allele Does Not Result in Skeletal Abnormalities

**FIGURE 2:**
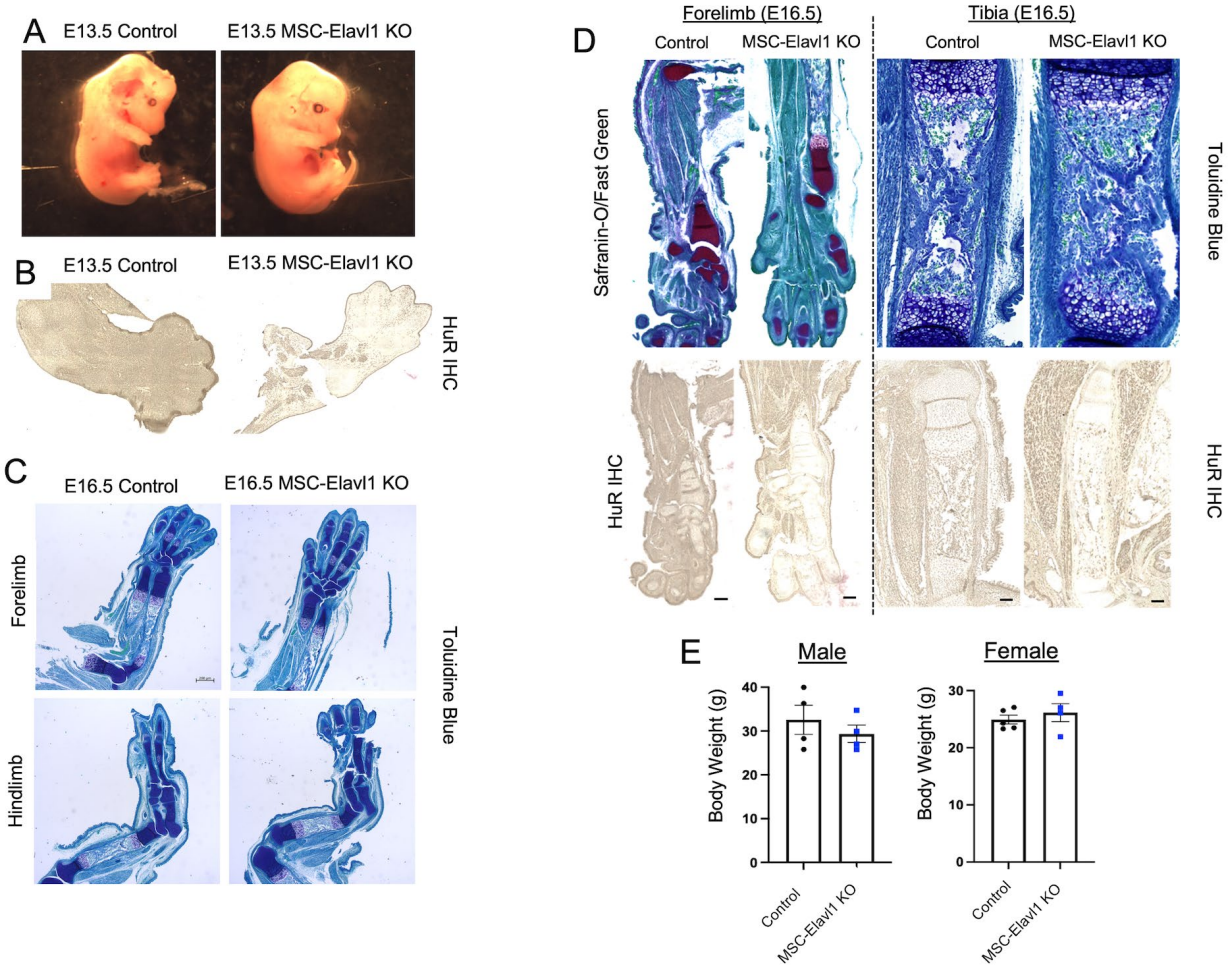
Effect of conditional knockout of HuR in limb mesenchyme through paternal inheritance of Prx1‐Cre in HuR^fl/fl^ mice. Control refers to HuR^fl/fl^ Prx1Cre^−/−^ and MSC‐Elavl1 KO refers to limb mesenchyme HuR^fl/fl^ Prx1Cre^+/−^ animals. (A) Micrographs of freshly dissected E13.5 embryos. (B) Immunohistochemical analysis of HuR protein in E13.5 embryo limbs. (C) Sections from E16.5 forelimbs and hindlimbs stained with toluidine blue. (D) Forelimbs and tibias of Control and MSC‐Elavl1 KO mice stained with safranin‐O/fast green (top panels) or immunohistochemistry using anti‐HuR (bottom panels). (E) Body weight in grams of male and female Control and MSC‐Elavl1 KO mice at 12 weeks of age.

By inhibiting expression of HuR in the developing mesenchyme through the use of paternally inherited Prx1‐Cre (subsequently referred to as MSC‐Elavl1KO), we aimed to reduce the impact of extrinsic developmental signaling and focus on how mesenchymal differentiation processes are influenced by HuR in the developing limb. Interestingly, we found that the embryos that inherited Cre paternally appeared similar to Control embryos, with no evidence of altered limb development (Figure 2A). Across four litters taken at E13.5, no embryos showed signs of skeletal growth retardation. Immunohistochemical analysis demonstrated that HuR protein was substantially depleted in mesenchyme‐derived tissues in E13.5 hindlimbs (Figure 2B). Patterning of distal hindlimb structures did not show any signs of disruption in Cre‐positive embryos at E16.5 (Figure 2C). Additionally, the structure of the endochondral growth plate in the femur at E16.5 did not appear to be disrupted by HuR knockdown (Figure 2D). Immunohistochemical analysis again demonstrated that HuR protein was depleted in the skeletal structures present by E16.5 (Figure 2D). We found that if the mice were allowed to develop, then they were able to grow to adulthood at a similar rate, with both males and females exhibiting similar body weights at 12 weeks of age (Figure 2E).

### 3.3 MSC‐Elavl1KO Does Not Result in Altered Articular Surface Integrity in Adult Mice

**FIGURE 3:**
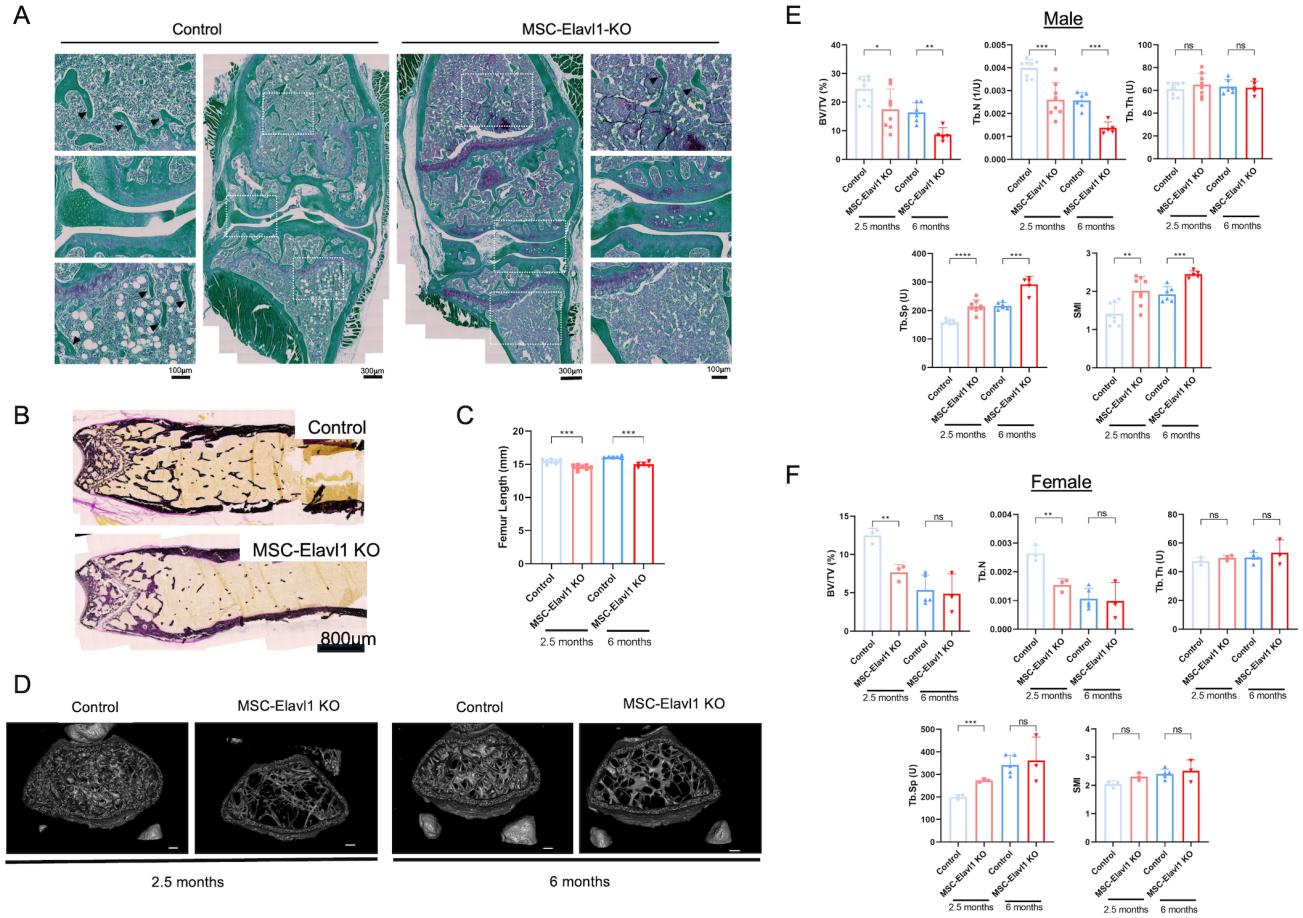
Analysis of bone structure and remodeling rate in adult mice following conditional HuR knockout in limb bud mesenchyme. (A) Safranin‐O/Fast Green‐stained sections of representative knee joints from 6‐month‐old male Control and MSC‐Elavl1KO mice, with higher magnification regions of areas indicated by dashed boxes. (B) Von Kossa‐stained, nondecalcified methylmethacrylate sections of the distal femur from Control and MSC‐Elavl1 KO mice. (C) Femur length determined from micro‐CT image data of Control and MSC‐Elavl1KO male mice. Individual data points presented with mean values +/− SD. Statistical analysis using an unpaired t‐test, ***p < 0.001. (D) Representative volumetric bone density visualizations of the proximal femur. Scale bar = 500 μm. Three‐dimensional bone morphometric parameters in (E) male and (F) female Control and MSC‐Elavl1 KO mice at 2.5 and 6 months of age. Percent bone volume density (BV/TV, %), trabecular Number (Tb.N, 1/U), trabecular separation (Tb.Sp, U), and structure model index (SMI). Individual data points presented with mean values +/− SD. ns *p* > 0.05, *p < 0.05, **p < 0.01, ***p < 0.001 t‐test comparisons between Control and MSC‐Elavl1KO.

To examine whether HuR is important for the maintenance of joint tissue homeostasis with age, we carried out histological analysis to examine the integrity of the articular cartilage in the knee joints at 6 months of age. We found that both MSC‐Elavl1KO and control mice exhibited no signs of joint pathology, indicating that impaired HuR expression in joint tissues does not predispose the joint to spontaneous arthritic changes (Figure 3A).

### 3.4 Disruption to Trabecular Bone Structure is Present in Adult MSC‐Elavl1KO Mice

Routine histological examination of the bones of 6‐month‐old male mice indicated that there was no effect of HuR knockout on the integrity of articular cartilage of the joints (Figure 3A) with low OARSI scoring (Male control 0+/−0, Male MSC‐Elavl1‐KO 0.16+/−0.21, Female Control 0.23 +/− 0.40, Female MSC‐ELavl1‐KO 0.1+/−0.17). However, we noted a marked loss of trabecular structure below the growth plate in the MSC‐Elavl1 KO animals (Figure 3A). We subsequently analyzed nondecalcified tissue, sectioned after embedding in methyl methacrylate, and stained with von Kossa stain. This very clearly demonstrated a loss of mineralized trabecular tissue in the femurs of the knockout animals at 2.5 months of age (Figure 3B). We analyzed micro‐CT scans of hindlimb tissues from the mice to quantify the loss of bone in the knockout animals. For the male mice, we performed lower resolution scans of the whole femur and found that HuR knockout resulted in a reduced length of the femurs of 2.5‐month‐old and 6‐month‐old male mice, by 0.87 (6%) and 1.07 mm (7%), respectively (Figure 3C). We used higher resolution scans to examine changes in bone structure in male and female mice. Figure 3D shows three‐dimensional reconstructions of the distal femurs of 2.5‐ and 6‐month‐old male MSC‐Elavl1 KO mice compared to controls. Loss of trabecular bone structure was observed in the femurs of the knockout mice and appeared to be more severe in the older animals. To quantify these changes, we performed three‐dimensional bone morphometric analysis of micro‐CT images from male and female MSC‐Elavl1 KO and control mice at the two age points (Figure 3E,F). We observed differences in bone microarchitecture in the male MSC‐Elavl1 KO mice at both 2.5 and 6 months of age: the HuR knockout decreased the percentage of trabecular bone volume to tissue volume (BV/TV) by 29% and 47%, respectively. This was due to 35% and 47% reduction in trabecular number (Tb.N), while trabecular thickness was not affected. The decrease in trabecular number resulted in a 35% increase in trabecular separation (Tb.Sp) at both ages, as well as decreased connectivity density (−35% at 2.5 months and −36% at 6 months). Increased structure model index (SMI, 43% and 27%, respectively) indicates a more rod‐like rather than plate‐like trabecular shape in the transgenics.

A similar decrease in trabecular bone was observed in female MSC‐Elavl1 KO mice at 2.5 months of age, with a 39% decrease in BV/TV and a 42% decrease in Tb.N, while again the trabecular thickness was not affected. Tb.Sp was increased by 37% in the 2.5‐month‐old knockout females, similar to the males. Although the SMI was increased, this was not statistically significant. Female mice showed a greater loss of bone structure in the controls from 2.5 to 6 months of age compared to the males. Probably because of the very low remaining bone volume, we could not detect a reduction in bone volume in the 6‐month‐old female mice.

Cortical bone parameters were assessed at the midshaft of the femur (Figure S1). Although cortical thickness was not affected, we found a change in bone size in the male transgenic mice at 6 months of age. Both the cross‐sectional tissue area and periosteal perimeter were reduced (by 20% and 10%, respectively). However, because the endosteal perimeter was also reduced (by 15%), the smaller periosteal perimeter had no effect on cortical thickness. The reduced bone size led to a substantial reduction in the moments of inertia along all major axes of approximately 30%. This indicates reduced resistance to bending in the transgenic mice. In addition, the polar moment of inertia was reduced by 29%, indicating a reduced resistance to torsion.

In conclusion, HuR knockout in the mesenchymal tissue of the limb results in impaired trabecular bone but not cortical bone structure in male mice and younger female mice. The effect on trabeculae was not cumulative, with increased bone volume loss observed naturally in females at 6 months of age.

### 3.5 Reduced Expression of HuR in Musculoskeletal Tissues Is Associated With a Reduction in the Distribution of Runx2 Protein

**FIGURE 4:**
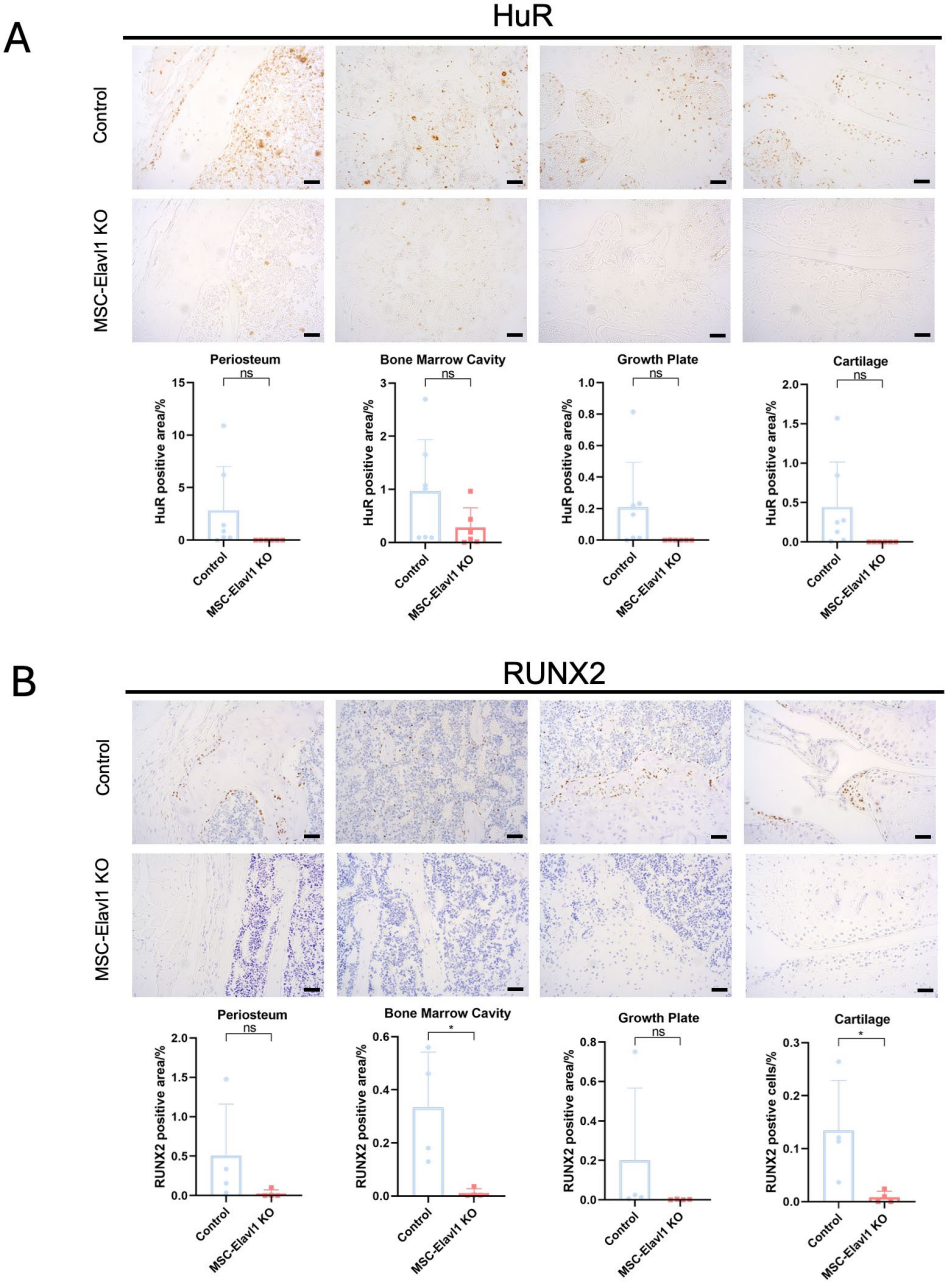
The distribution of the osteoblast marker Runx2 protein is reduced in MSC‐Elavl1 KO mice mouse skeletal tissues. (A) Immunohistochemical analysis of HuR from Control and MSC‐Elavl1 KO mice with representative images and stained area quantification (Control: *N* = 7, MSC‐Elavl1 KO: *N* = 6). (B) Immunohistochemistry analysis of RUNX2 from Control and MSC‐Elavl1 KO mice with representative images and stained area quantification (*n* = 4). All scale bars in the figure = 20 μm. In all charts, Control refers to HuR^fl/fl^ Prx1Cre^−/−^ and MSC‐Elavl1 KO refers to HuR^fl/fl^ Prx1Cre^+/−^.

We investigated the distribution of HuR protein in the limb tissue of 6‐month‐old control and MSC‐Elavl1KO mice. Using immunohistochemistry with subsequent image analysis, we measured the proportion of area stained for HuR in our sections (Figure 4A). As expected, HuR expression was reduced in the skeletal tissues of the knockout animals. Because of the effect HuR had on bone density, we also examined the protein distribution of the osteoblast marker Runx2 (Figure 4B) and found that its abundance was also reduced in the knockout animals, most strikingly in the bone marrow cavity and the articular cartilage.

### 3.6 Osteoblasts Isolated From MSC‐Elavl1 KO Mice Have an Impaired Capacity to Form Mineralized Extracellular Matrix

**FIGURE 5:**
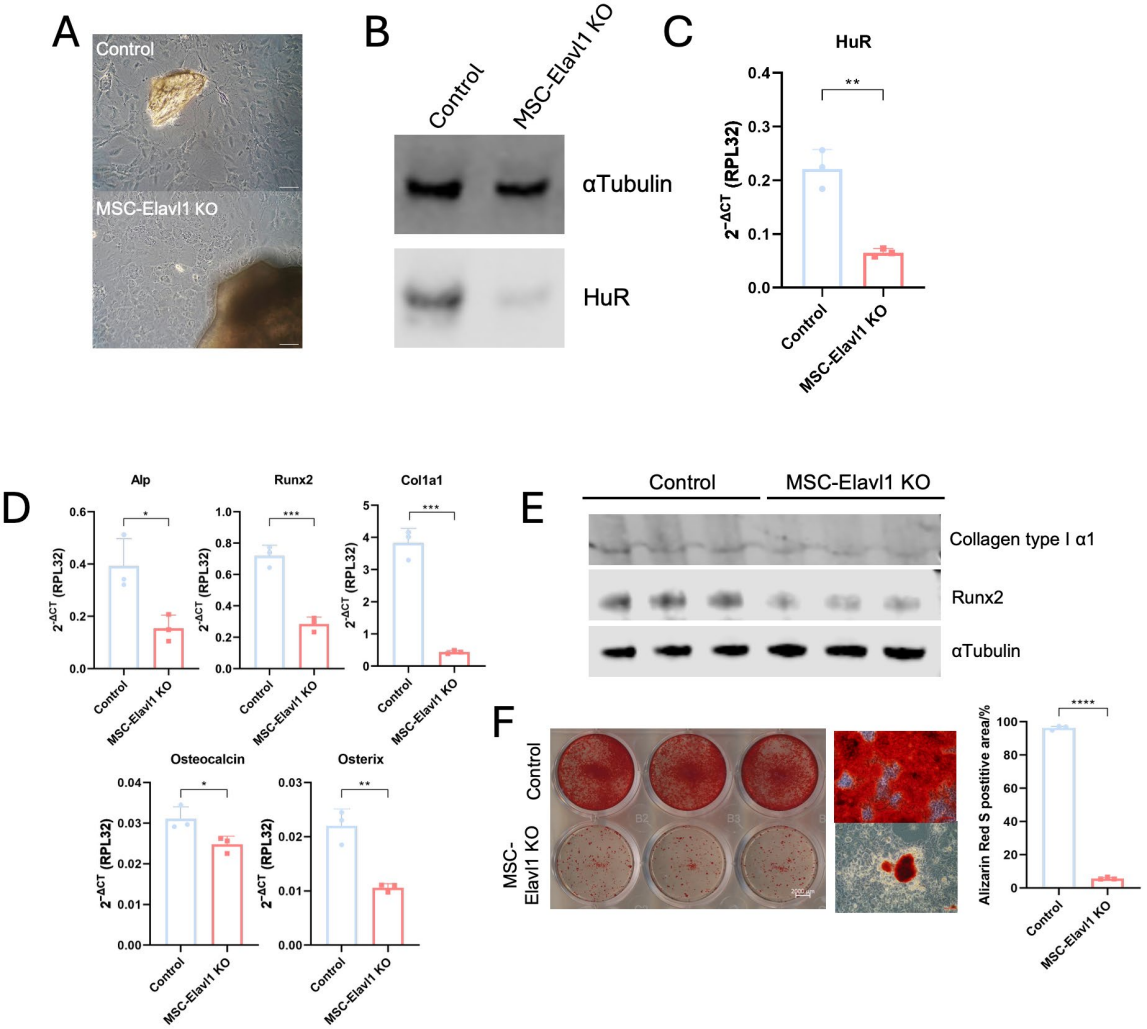
Isolation and analysis of osteoblasts isolated from 2.5‐month‐old Control and MSC‐Elavl1 KO mice. (A) Phase micrographs of primary osteoblasts isolated from long bone chips. Scale bar = 100um. (B) Western blotting and (C) RT‐qPCR analysis of HuR protein and transcript levels in isolated osteoblasts. (D) RT‐qPCR analysis of osteoblast marker genes in isolated cells. (E) Western blot analysis of protein levels of Collagen type I alpha 1 and Runx2 proteins in isolated osteoblasts. (F) Alizarin Red S staining of primary osteoblasts following 21 days in mineralization conditions. The left panel shows staining of the whole 6‐well plate with Control cells on the top row and MSC‐Elavl1 KO cells on the bottom row. The middle panel shows a higher magnification of alizarin red staining of the Control and MSC‐Elavl1 KO cell cultures. Right‐most panel shows quantification of solubilized Alizarin Red stain from Control and MSC‐Elavl1 KO cultures.

Our analysis had identified bone as a tissue that was critically affected by Prx1‐Cre‐mediated loss of HuR in the limb mesenchymal tissue. Therefore, we undertook an in vitro analysis to better understand the effect of HuR on bone biology. Primary osteoblasts were isolated from the long bones of MSC‐Elavl1KO or control mice (Figure 5A). Western blot and RT‐qPCR verified that HuR protein and exon 2‐containing HuR mRNA were strongly reduced in the knockout primary osteoblasts (Figure 5B,C). This loss of HuR expression coincided with reduced mRNA expression of the osteoblast‐marker genes ALP (reduced to 0.39‐fold), RUNX2 (reduced to 0.39‐fold), Col1a1 (reduced to 0.11‐fold), Osteocalcin (reduced to 0.80‐fold), and Osterix (reduced to 0.48‐fold) (Figure 5D). We confirmed regulation of RUNX2 and collagen type I, two of the most strongly regulated marker genes, at the protein level (Figure 5E), observing consistent reduction in their protein levels in different MSC‐Elavl1 KO cell cultures. To assess the impact of the loss of HuR on osteoblast function, their capacity to synthesize a mineralized extracellular matrix was measured using Alizarin Red S staining after a 21‐day exposure to mineralizing culture conditions (Figure 5F). The ability of the MSC‐Elavl1 KO osteoblasts to produce a mineralized matrix was substantially lower than the cells from control mice. This in vitro analysis demonstrated that loss of HuR strongly affects osteoblasts' phenotype and ability to produce extracellular matrix.

### 3.7 HuR Knockout in Osteoblasts Results in Substantial Changes in Transcript Levels

**FIGURE 6:**
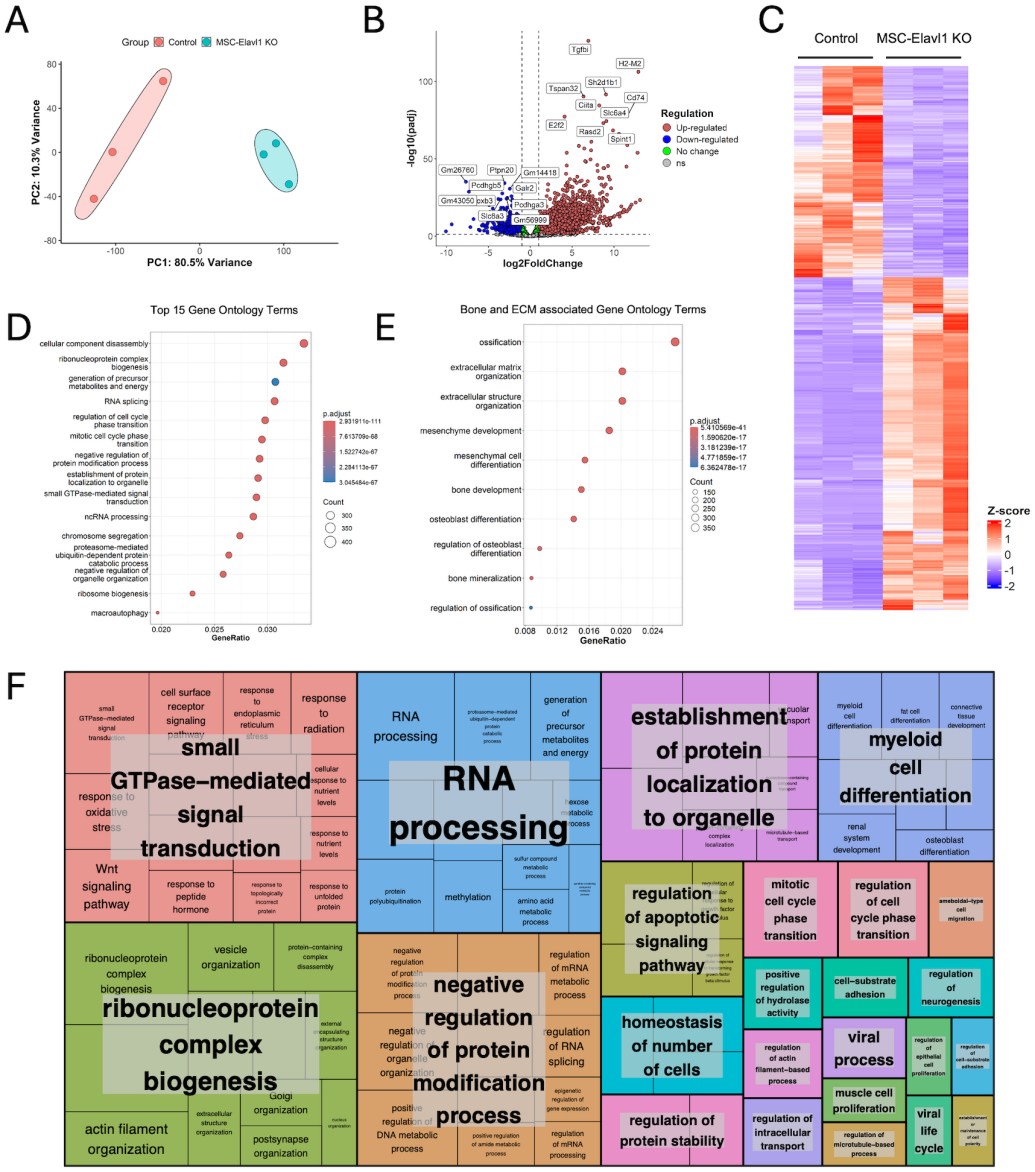
RNA‐Seq analysis of transcript expression in osteoblasts isolated from 2.5‐month‐old Control and MSC‐Elavl1KO mice. (A) Principal components analysis, (B) volcano plot, and (C) heat map illustrating transcriptome variation identified by DeSeq2 analysis of reads obtained from osteoblasts isolated from 2.5‐month‐old Control and MSC‐Elavl1 KO mice. DeSeq2 analysis of RNA‐seq. (D) Top 15 ontology terms and (E) bone and extracellular matrix (ECM)‐related ontology terms, identified by gene set enrichment analysis following DeSeq2 analysis of RNA‐Seq data from isolated osteoblasts. (F) REVIGO generated a treemap summarization of nearly 2000 enriched gene ontology terms identified by the gene set enrichment analysis, grouped by semantic similarity. Box size is proportional to ‐log10 *p* value. Box color represents gene ontology term cluster, highlighted label = representative term.

We wanted to understand the wider impact of HuR loss on the osteoblast transcriptome, so we performed RNA‐Seq analysis on total RNA isolated from MSC‐Elavl1 KO and control osteoblast cultures. We observed a substantial degree of variance in the transcript expression levels between the two populations, illustrated through principal component analysis (Figure 6A). The differential expression analysis identified 3312 transcripts that exhibited a more than two‐fold change in expression, with an adjusted *p* value of < 0.05 in the knockout osteoblasts (Figure 6B,C and Table S2). Gene enrichment analysis associated nearly 5000 GO terms, and those with the lowest adjusted *p* values are shown in Figure 6D. We focused on bone and extracellular matrix‐related processes and found that genes enriched with these processes were identified by the analysis (Figure 6E). To summarize the GO terms in a nonbiased way, we used REVIGO, which generated representative sets, grouped by semantic similarity, from the top 2000 identified GO terms (Figure 6F). We found that the strongest associations of HuR‐regulated genes are with processes linked to RNA processing, ribonucleoprotein complex biogenesis, small GTPase‐mediated cell transduction, regulation of apoptotic signaling, protein localization, and myeloid cell signaling, among others. By studying the transcriptome‐wide effects of HuR knockout in osteoblasts, it is clear that a wide range of cell processes are impacted, with links both to well‐understood bone biology as well as wider cellular functions.

### 3.8 HuR Knockdown Alters mRNA Decay of RUNX2 in Human Osteoblastic Cells

**FIGURE 7:**
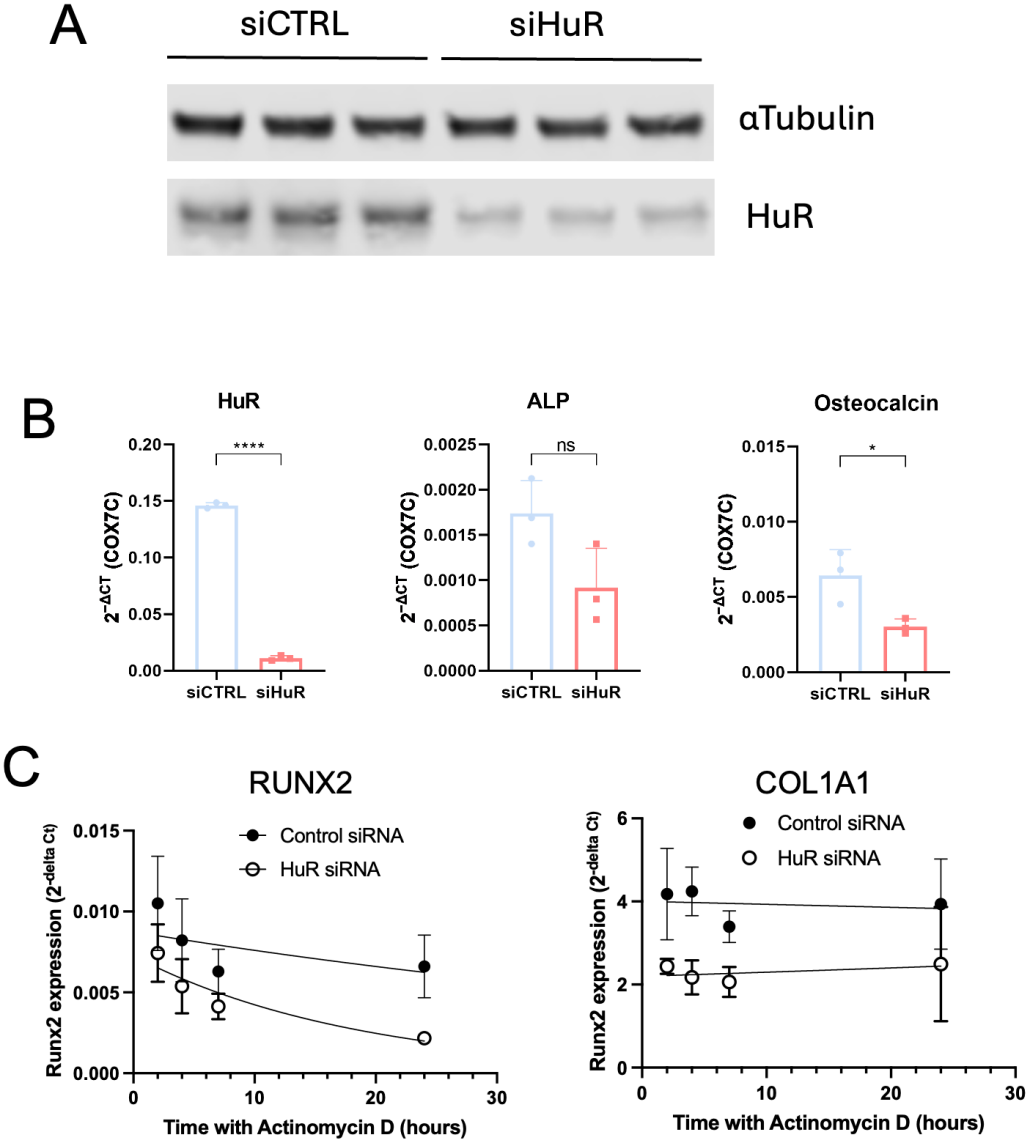
Analysis of siRNA‐mediated HuR knockdown in MG63 cells. (A) Western blot analysis of HuR and αTubulin protein levels following siRNA treatment. (B) RT‐qPCR quantification of HuR and bone marker gene expression in siRNA‐treated MG63 cells. (C) RT‐qPCR analysis of RUNX2 and COL1A1 mRNA decay in siRNA‐treated cells from the Actinomycin D chase experiment.

To examine the effect of HuR loss in a human osteoblastic lineage cell, we used the human MG63 osteosarcoma cell line as a model system and transfected the cells with siRNA targeting HuR mRNA. Using subsequent western blot and RT‐qPCR analysis, we found that a 72‐h period of transfection led to a strong downregulation of HuR protein and mRNA compared to nonspecific siRNA‐transfected controls (Figure 7A,B). We used this system to examine how transcript levels of osteoblast marker genes were affected and found that HuR knockdown cells exhibited reduced levels of ALP and Osteocalcin, consistent with our observations in the murine knockout primary osteoblasts (Figure 7B). We expanded our analysis to examine two other markers, RUNX2 and COL1A1, by assessing their expression levels and using an actinomycin D chase to determine their mRNA half‐life. We found that the mRNA of both was expressed at lower levels in HuR knockdown MG63 cells, again, consistent with our findings in the murine system (Figure 7C). Furthermore, RUNX2 mRNA was turned over quickly in HuR knockdown cell cultures with a mean control siRNA of over 24 h and a mean HuR siRNA half‐life of 14.4 h. COL1A1 mRNA was very stable in control siRNA‐treated cells (> 24‐h half‐life), and this was not affected by the HuR knockdown.

## 4 Discussion

This study aimed to determine the role of HuR in the development and function of musculoskeletal tissues of the limb. Using the Prx1‐Cre conditional knockout system [16] with a previously described HuR^fl/fl^ mouse line [13], we were able to drive knockout of HuR in developing limb bud mesenchyme and showed defective skeletal formation. Previous epiblast‐specific conditional knockout in the same floxed mouse line led to a disruption of limb skeletal development [13], and we had expected that disruption of HuR in the early mesenchyme of the limb would at least partially replicate this. However, we have found that limb mesenchyme expression of HuR is not necessary for limb development and growth in mice. Instead, we have shown that the lack of HuR in limb musculoskeletal tissues manifests itself through a reduction in trabecular bone density in adult animals, identifying a previously unconfirmed role for it in the control of bone remodeling.

An established property of the Prx1‐Cre system is that a limb mesenchyme‐specific conditional knockout must be driven through paternal inheritance of the Cre allele [16]. Maternal inheritance can result in germline transmission of the Cre allele. We initially used this latter property to generate a widespread HuR knockout, and we found evidence of the same developmental retardation seen in the Sox2‐driven epiblast knockouts [13], including abnormal limb growth. Based upon the Sox2‐driven study, we would anticipate that our Prx1‐Cre‐mediated germline knockout is ultimately embryonically lethal, although we did not directly examine this. In contrast, we found that conditional knockout in limb mesenchyme driven by paternal inheritance of the Cre allele did not generate any notable skeletal phenotype and that the mice could develop to term healthily and grow to adulthood. This finding suggests that the skeletal defects observed when HuR is knocked out in the epiblast are driven by signals generated by tissues outside of the condensing limb mesenchyme. Through further analysis of maternally inherited Prx1‐Cre embryos using in situ HCR, we found that there is disruption to the expression of Fgf8 by cells within the apical ectodermal ridge in limb buds of HuR knockouts. Signaling from the AER [25, 26] is critically important for controlling the proximodistal axis of the growing limb bud, with disruption to this signaling process having well‐established links to malformation of the limb skeleton [27]. Interestingly, another classical regulator of limb patterning, the ZPA [28, 29], which is found in the posterior region of the developing limb mesenchyme and visualized using the expression of Shh, was still detectable in the HuR knockout embryos.

Analysis of the conditional HuR knockout in limb mesenchyme demonstrated a subtle effect on limb development overall, with a small reduction in the femur length of knockout animals. Detailed study of the bones of adult mice through histological staining and micro‐CT scanning also showed that there was a substantial reduction in the trabecular structure of the long bones when HuR expression was disrupted, manifesting itself through reduced trabecular volume and number with an increase in trabecular separation. As expected, many tissues in the limb skeleton had markedly reduced HuR protein expression in the knockout mice. We were able to show that there was also disruption to the osteoblast marker Runx2, as well as in tissues such as the bone marrow cavity and the articular cartilage. Bone remodeling is a highly dynamic process driven by bone deposition by mesenchyme‐derived osteoblasts and bone resorption by osteoclasts, which have hematopoietic origins. Reduced HuR expression has been associated with loss of bone density in ovariectomized mice [30, 31], and inhibition of HuR in osteoblast cell lines can reduce their osteogenic capacity with associated loss of expression of markers such as Runx2. This study builds upon the associations identified in the previous studies, demonstrating through gene knockout in vivo that HuR performs an important role in bone homeostasis.

There have been several studies investigating the role of HuR in bone homeostasis; however, their findings remain controversial. Some studies have shown that HuR is positively correlated with osteogenic differentiation, suggesting that increased HuR expression could help mitigate osteoporosis [30, 31, 32, 33]. In contrast, Ren and colleagues reported that knocking down HuR may alleviate osteoporosis by regulating divalent metal transporter 1 (DMT1) under diabetic conditions [34]. Our study shows that HuR has a strong influence on osteoblast transcript levels and on their ability to produce a mineralized extracellular matrix. Expression of marker genes such as Runx2 [35], osteocalcin, and osterix is consistent with this. Previous studies have linked HuR expression to the capacity of stem cells to differentiate into osteoblasts [32]. Measurement of wider transcriptomic changes using RNA‐Seq reveals that the principal processes associated with genes affected by HuR knockout are linked to a wide variety of processes. These range from control of RNA and ribonucleoprotein function to cell division, cell death, and metabolism. Among the pathways identified were also many highly relevant to bone biology, including tissue mineralization, extracellular matrix organization, and bone cell differentiation. Overall, the breadth of processes that appear to be potentially affected by HuR in osteoblasts indicates that further understanding of the underlying mechanisms could help us to understand more about already established bone pathways, as well as provide insights into how bone biology is affected in processes where there has been less focus.

In conclusion, this study provides strong evidence of a key role for HuR in the maintenance of bone health by mesenchyme‐derived cells. On top of the recent report identifying HuR as a potential regulator of bone hemostasis in osteoporosis models [30], our findings further underscore the potential for studying how HuR‐mediated processes could be targeted to reduce the impact of loss of bone density associated with aging and frailty. Our study also shows that despite the importance of HuR expression in bone in the adult mice, development of skeletal tissues in the limb does not rely on their own expression of HuR and that its expression in extrinsic structures, such as the AER, must be critical for appropriate signaling of mesenchymal differentiation during limb development.

## Author Contributions

S.F., K.A.J., P.W., U.E., and RJVH contributed to the design and conduction of the study. B.T.M., C.K., and I.K. contributed to experiments; A.V., D.K., PM, and D.A.T. contributed to the discussion and interpretation of data. S.R.T. and G.B. conceived the project, designed, and supervised the experiments. All authors revised the manuscript and approved the final version.

## Conflicts of Interest

The authors declare no conflicts of interest.

## Supporting information


**Figure S1:** Three‐dimensional bone morphometric and biomechanical parameters relating to cortical bone structure in male and female Control and MSC Elavl1 KO mice at 2.5 and 6 months of age. Cortical Thickness (Cort.Th, 1/U), Mean total cross‐sectional tissue area (T.Ar, U2), Periosteal perimeter (Per.Pm, U), Endosteal perimeter (End.Pm, U), Average moment of inertia (x) (Av.MMI (x), U4), Average moment of inertia (y) (Av.MMI (y), U4), Mean polar moment of inertia (MMI (polar), U4). Individual data points presented with mean values +/− SD. ns = *p* > 0.05, t‐test comparisons between Control and MSC‐Elavl1KO.

**Table S1:** Sequences of HCR v3 split initiators, probe sets, and corresponding amplifiers for Fgf8 and Shh.

**Table S2:** DeSeq2 differential expression analysis of the transcriptome of osteoblasts isolated from 2.5‐month‐old Control and MSC‐Elavl1KO mice. Genes were filtered with the following thresholds: baseMean > 50, padj < 0.05, log2FoldChange > 1 or < −1 and the 50 genes with the lowest adjusted *p* values are presented.

## Data Availability

As described in the materials and methods, transcriptomic analysis read data contained in trimmed FastQ files have been deposited on ArrayExpress with the accession number E‐MTAB‐14888 (https://www.ebi.ac.uk/biostudies/arrayexpress/studies/E‐MTAB‐14888). Other datasets used during the study are available from the corresponding author on reasonable request.
