# Supplementary material for: Loss of the RNA Binding Protein HuR in Early Murine Limb Mesenchyme Does Not Affect Development but Leads to Impaired Bone Homeostasis in Adulthood
Source: FASEB J. 2025 Nov 20;39(22):e71222. doi: 10.1096/fj.202500780RR (PMC12631158; doi:10.1096/fj.202500780RR)

Supplementary Figure 1: Three-dimensional bone morphometric and biomechanical parameters relating to cortical bone structure in male and female Control and MSC-Elavl1 KO mice at 2.5- and 6-months of age. Cortical Thickness (Cort.Th, 1/U), Mean total crossectional tissue area (T.Ar, U^2), Periosteal perimeter (Per.Pm, U), Endosteal perimeter (End.Pm, U), Average moment of inertia (x) (Av.MMI(x), U^4), Average moment of inertia (y) (Av.MMI(y), U^4), Mean polar moment of inertia (MMI(polar), U^4). Individual data points presented with mean values +/- SD. ns = P > 0.05, t-test comparisons between Control and MSC-Elavl1KO.

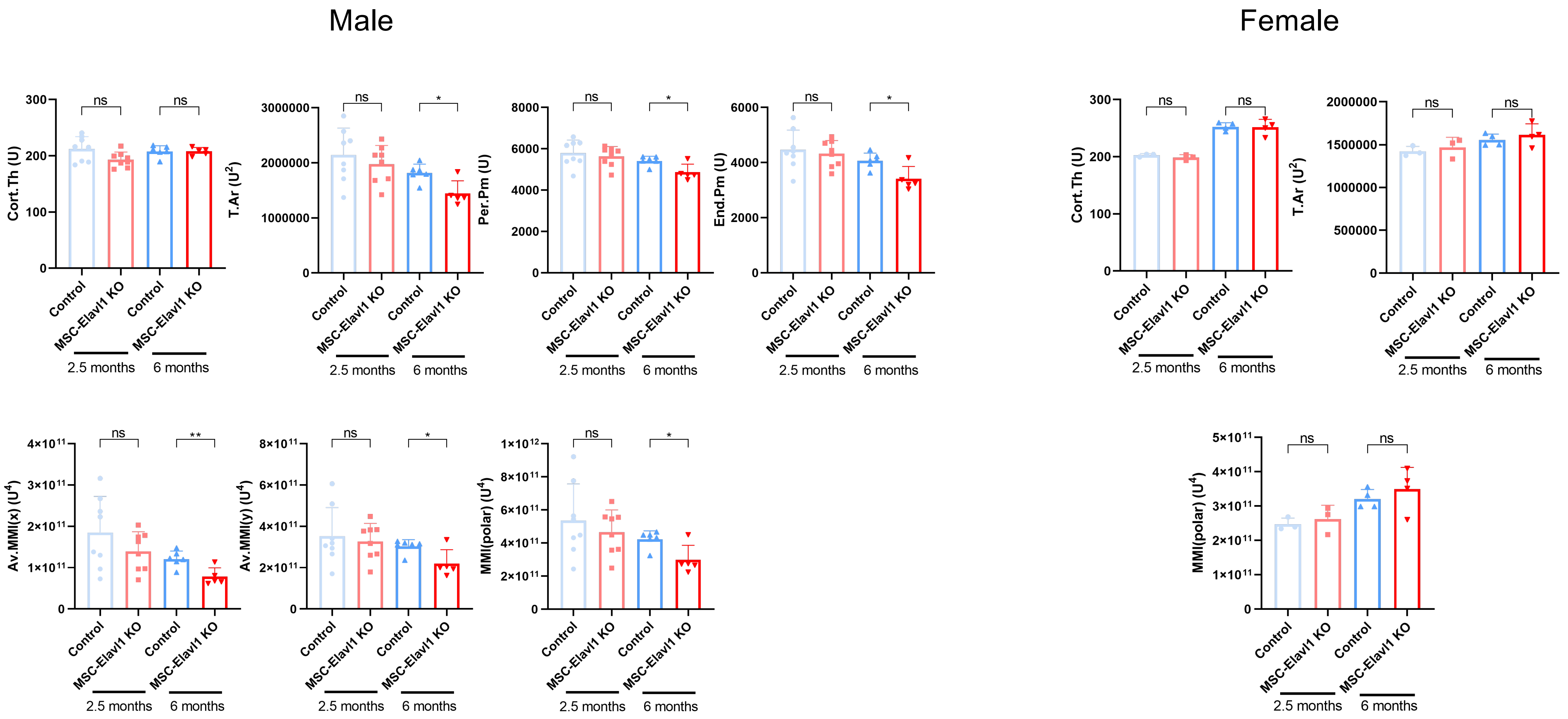

Supplement: Supplementary file 1 — Figure S1: Three‐dimensional bone morphometric and biomechanical parameters relating to cortical bone structure in male and female Control and MSC Elavl1 KO mice at 2.5 and 6 months of age. Cortical Thickness (Cort.Th, 1/U), Mean total cross‐sectional tissue area (T.Ar, U2), Periosteal perimeter (Per.Pm, U), Endosteal perimeter (End.Pm, U), Average moment of inertia (x) (Av.MMI (x), U4), Average moment of inertia (y) (Av.MMI (y), U4), Mean polar moment of inertia (MMI (polar), U4). Individual data points presented with mean values +/− SD. ns = p > 0.05, t‐test comparisons between Control and MSC‐Elavl1KO. [file FSB2-39-e71222-s003.pdf]
