# Supplementary material for: Loss of the RNA Binding Protein HuR in Early Murine Limb Mesenchyme Does Not Affect Development but Leads to Impaired Bone Homeostasis in Adulthood
Source: FASEB J. 2025 Nov 20;39(22):e71222. doi: 10.1096/fj.202500780RR (PMC12631158; doi:10.1096/fj.202500780RR)
Supplement: Supplementary file 2 — Table S1: Sequences of HCR v3 split initiators, probe sets, and corresponding amplifiers for Fgf8 and Shh. [file FSB2-39-e71222-s002.docx]

**Supplementary Table 1:** Sequences of HCR v3 split initiators, probe sets and corresponding amplifiers for Fgf8 and Shh.

**Organism: mus musculus, Target mRNA: Fibroblast growth factor 8 (Fgf8), Probe set: 10 split-initiator probe pairs (each probe carries half an HCR initiator)**, **HCR amplifier: B3-Alexa594**

|  | Initiator I1 | Probe sequence | Spacer | Probe sequence | Initiator I2 |
| --- | --- | --- | --- | --- | --- |
| **1** | gtccctgcctctatatcttt | TGGAGCCGGTGGCCGCTGGCTGCTC | GC | GCGCGCCGAACCCCTCTGAGCCGCT | ttccactcaactttaacccg |
| **2** | gtccctgcctctatatcttt | GGGACAAGCCGAAGGTGCGGAGGCT | GG | TCACGCCGTCCCACTGGAGGCCGCG | ttccactcaactttaacccg |
| **3** | gtccctgcctctatatcttt | AGAGAACCAGCAAGTGCAACAGCAG | GC | CGCCCGGGCCTTCCTGGGCTTGGAG | ttccactcaactttaacccg |
| **4** | gtccctgcctctatatcttt | GTGAGGACTGAACAGTTACCTGTTG | AG | GCTCCCTCACATGCTGTGTAAAATT | ttccactcaactttaacccg |
| **5** | gtccctgcctctatatcttt | TGGCCAGGACCTGCACGTGCTTCCC | GT | CTTCTGCCATGGCGTTGATGCGCTT | ttccactcaactttaacccg |
| **6** | gtccctgcctctatatcttt | TGCAGATGTAGAGACCTGTCTCTGC | CA | TGGCAATTAGCTTCCCCTTCTTGTT | ttccactcaactttaacccg |
| **7** | gtccctgcctctatatcttt | ACCAGCCCTCGTACTTGGCGTTCTG | GT | GCCGGCCCTTGCGGGTAAAGGCCAT | ttccactcaactttaacccg |
| **8** | gtccctgcctctatatcttt | GCAGGCTCTGCTCGGTGGTGTGGTG | GC | AGGGCGGGTAGTTGAGGAACTCGAA | ttccactcaactttaacccg |
| **9** | gtccctgcctctatatcttt | ATTCCTCGGCCGGCTGGGTGGGGAG | GG | TTTGCTGTGCCGCCGAGCTCCCGCT | ttccactcaactttaacccg |
| **10** | gtccctgcctctatatcttt | CCTCCACCCCAGGGAACCCCAGAGG | GC | GGATTTCAGGAGAACAGACCAGAGA | ttccactcaactttaacccg |

**Organism: mus musculus, Target mRNA: Sonic hedgehog (Shh), Probe set: 20 split-initiator probe pairs (each probe carries half an HCR initiator), HCR amplifier: B4-Alexa647**

|  | Initiator I1 | Probe sequence | Spacer | Probe sequence | Initiator I2 |
| --- | --- | --- | --- | --- | --- |
| **1** | cctcaacctacctccaacaa | GTCTCGAGACAGCAATTAAAAGACA | GG | GCTGGTAACGGAACACATCGGAGTT | attctcaccatattcgcttc |
| **2** | cctcaacctacctccaacaa | GGTGTGTGCGTGTGCGCTCCTCCTT | CG | CTGTCTGTGCGCGAGCGGGTACGCG | attctcaccatattcgcttc |
| **3** | cctcaacctacctccaacaa | TGTAGGCTAAAGGGGTCAGCTTTTT | CT | TCTCGGCTACGTTGGGAATAAACTG | attctcaccatattcgcttc |
| **4** | cctcaacctacctccaacaa | CGGACGTGGTGATGTCCACTGCTCG | GT | GCATGCCGTACTTGCTGCGGTCCCG | attctcaccatattcgcttc |
| **5** | cctcaacctacctccaacaa | AGCACGCGGTCTCCGGGACGTAAGT | CC | AGCAGCCGGCCCTGGTCGTCAGCCG | attctcaccatattcgcttc |
| **6** | cctcaacctacctccaacaa | TGCACCGCGGCGGGCAGCAGCCGGC | TG | GCCTCCTCCTCTCGCAGCGTCACGC | attctcaccatattcgcttc |
| **7** | cctcaacctacctccaacaa | AGGCCCGGTGTGCCCAGCTGTGCTC | GA | GCGCGTGCGCCAGGCGGAAAGGCGC | attctcaccatattcgcttc |
| **8** | cctcaacctacctccaacaa | CCAGTGGATGCCCGCAGTCGGCTCC | TA | GCCAATGTGGTAGAGCAGCTGCGAG | attctcaccatattcgcttc |
| **9** | cctcaacctacctccaacaa | TATTTCGCAGTCGCTCCCCGCCCCG | CT | CTTCCGTGCGCTTTCCCATCAGTTC | attctcaccatattcgcttc |
| **10** | cctcaacctacctccaacaa | TCTGCTCCCGTGTTTTCCTCATCCT | GG | TCTTTGCACCTCTGAGTCATCAGCC | attctcaccatattcgcttc |
| **11** | cctcaacctacctccaacaa | GCTTCCTTATAGTCTACTTTGGACT | TT | ATAACAACAGAACTCCCCGGGGTTT | attctcaccatattcgcttc |
| **12** | cctcaacctacctccaacaa | TTCTGAAACGCAGGACAAGGGACAT | CC | AGCAGGAGAGGAATGCGGAGGTTTG | attctcaccatattcgcttc |
| **13** | cctcaacctacctccaacaa | AGCAAATACAAACCAAGAAGGCAGT | AA | AAGAACATGACAAAGTGGCGGTTAC | attctcaccatattcgcttc |
| **14** | cctcaacctacctccaacaa | ATCGTTCGGAGTTTCTTGTGATCTT | AA | GGTTGTAATTGGGGGTGAGTTCCTT | attctcaccatattcgcttc |
| **15** | cctcaacctacctccaacaa | TTCATAGTAGACCCAGTCGAAACCT | GA | CACAGAACAGTGGATGTGAGCTTTG | attctcaccatattcgcttc |
| **16** | cctcaacctacctccaacaa | CTCGATCACGTAGAAGACCTTCTTG | GT | CAGCAGGCGCTCGCGCGGCTCCAGC | attctcaccatattcgcttc |
| **17** | cctcaacctacctccaacaa | CGTTGTGCGGCGCCACGAAGAGCAG | GT | CGCTTGGCCCGGGCGTGGGCCCCGA | attctcaccatattcgcttc |
| **18** | cctcaacctacctccaacaa | GCCGGATTTGGCCGCCACGGAGTTC | CC | CACGGTGGCGGATCCCGGGAAACAG | attctcaccatattcgcttc |
| **19** | cctcaacctacctccaacaa | TGCCGTGCGCCGTGAGCGGCGCGTA | GG | AGGCGAGCACCCGGTTGATGAGAAT | attctcaccatattcgcttc |
| **20** | cctcaacctacctccaacaa | TGGACTTGACCGCCATTCCCAAGGG | GC | CCTTGCCCGGTCCCGTCGGGCTTCA | attctcaccatattcgcttc |
