# Supplementary material for: Loss of the RNA Binding Protein HuR in Early Murine Limb Mesenchyme Does Not Affect Development but Leads to Impaired Bone Homeostasis in Adulthood
Source: FASEB J. 2025 Nov 20;39(22):e71222. doi: 10.1096/fj.202500780RR (PMC12631158; doi:10.1096/fj.202500780RR)
Supplement: Supplementary file 3 — Table S2: DeSeq2 differential expression analysis of the transcriptome of osteoblasts isolated from 2.5‐month‐old Control and MSC‐Elavl1KO mice. Genes were filtered with the following thresholds: baseMean > 50, padj < 0.05, log2FoldChange > 1 or < −1 and the 50 genes with the lowest adjusted p values are presented. [file FSB2-39-e71222-s001.docx]

**Supplementary Table 2:** DEseq2 differential expression analysis of transcriptome of osteoblasts isolated from 2.5-month-old Control and MSC-Elavl1KO mice. Genes were filtered with following thresholds: baseMean > 50, padj < 0.05, log2FoldChange > 1 or < -1 and the 50 genes with lowest adjusted p values are presented.

| ENSEMBL_ID | baseMean | log2FoldChange | padj | Gene_name |
| --- | --- | --- | --- | --- |
| ENSMUSG00000035493 | 36374.15194 | 6.941305204 | 5.26E-127 | Tgfbi |
| ENSMUSG00000016283 | 19537.7825 | 12.90271699 | 5.66E-107 | H2-M2 |
| ENSMUSG00000102418 | 3246.235998 | 9.038789383 | 2.46E-92 | Sh2d1b1 |
| ENSMUSG00000000244 | 1263.748324 | 6.362491224 | 4.98E-91 | Tspan32 |
| ENSMUSG00000022504 | 6834.886887 | 8.233847155 | 3.36E-85 | Ciita |
| ENSMUSG00000024610 | 348059.8384 | 11.56497578 | 3.62E-78 | Cd74 |
| ENSMUSG00000018983 | 2600.849267 | 4.101755931 | 5.31E-78 | E2f2 |
| ENSMUSG00000020838 | 4654.295207 | 9.091433558 | 4.22E-75 | Slc6a4 |
| ENSMUSG00000034472 | 1798.842704 | 8.731931878 | 7.92E-74 | Rasd2 |
| ENSMUSG00000027315 | 1448.470743 | 9.884384578 | 4.48E-69 | Spint1 |
| ENSMUSG00000030142 | 8669.858779 | 10.59870835 | 7.85E-67 | Clec4e |
| ENSMUSG00000054423 | 2058.860506 | 9.743009945 | 7.68E-63 | Cadps |
| ENSMUSG00000029189 | 962.20105 | 7.292694009 | 8.75E-62 | Sel1l3 |
| ENSMUSG00000025804 | 2593.738715 | 9.873489347 | 1.36E-61 | Ccr1 |
| ENSMUSG00000060586 | 103276.503 | 11.58853884 | 1.41E-59 | H2-Eb1 |
| ENSMUSG00000026068 | 931.6878423 | 5.802898382 | 4.09E-59 | Il18rap |
| ENSMUSG00000050138 | 786.6334802 | 6.44914445 | 2.12E-57 | Kcnk12 |
| ENSMUSG00000029648 | 4337.872426 | 7.260129138 | 3.57E-56 | Flt1 |
| ENSMUSG00000024053 | 16405.48749 | 4.364724815 | 1.21E-54 | Emilin2 |
| ENSMUSG00000036594 | 135846.4668 | 12.81526314 | 1.21E-54 | H2-Aa |
| ENSMUSG00000051457 | 6529.905819 | 7.195093361 | 2.39E-50 | Spn |
| ENSMUSG00000118330 | 615.9305066 | 6.56198265 | 3.17E-49 | Gm30042 |
| ENSMUSG00000001588 | 485.3307684 | 6.644799427 | 8.65E-49 | Acap1 |
| ENSMUSG00000121619 | 483.8666299 | 8.006394581 | 9.53E-47 | 5430437J10Rik |
| ENSMUSG00000025422 | 426.1469731 | 4.364724707 | 2.09E-46 | Agap2 |
| ENSMUSG00000028362 | 1167.801963 | 9.28186056 | 4.34E-46 | Tnfsf8 |
| ENSMUSG00000073421 | 104921.2615 | 11.48631644 | 1.1E-45 | H2-Ab1 |
| ENSMUSG00000019852 | 1849.421757 | 8.735468649 | 9.36E-45 | Arfgef3 |
| ENSMUSG00000003882 | 6690.656029 | 8.823426671 | 1.06E-44 | Il7r |
| ENSMUSG00000035314 | 1954.391865 | 2.447747569 | 1.02E-43 | Gdpd5 |
| ENSMUSG00000086564 | 450.9679346 | 8.049980127 | 7.41E-43 | Cd101 |
| ENSMUSG00000018500 | 246.6154721 | 4.278834353 | 4.47E-41 | Adora2b |
| ENSMUSG00000020788 | 1907.860378 | 5.613834169 | 3.43E-39 | Atp2a3 |
| ENSMUSG00000022303 | 1922.171355 | 9.344309702 | 3.88E-39 | Dcstamp |
| ENSMUSG00000041538 | 842.7230667 | 8.246083653 | 2.26E-38 | H2-Ob |
| ENSMUSG00000058427 | 723.7954865 | 8.586264932 | 3.27E-38 | Cxcl2 |
| ENSMUSG00000025161 | 6480.316231 | 4.741975513 | 1.34E-37 | Slc16a3 |
| ENSMUSG00000102715 | 873.5101536 | 9.500336332 | 2.42E-37 | Gm6209 |
| ENSMUSG00000030344 | 220.2145903 | 5.257922067 | 3.87E-37 | Akap3 |
| ENSMUSG00000020798 | 284.9994447 | 5.323508073 | 1.33E-36 | Spns3 |
| ENSMUSG00000044708 | 389.8395581 | 6.137740028 | 1.33E-36 | Kcnj10 |
| ENSMUSG00000062082 | 1262.150742 | 6.865709878 | 3.33E-36 | Cd200r4 |
| ENSMUSG00000031391 | 14633.8813 | 5.96366226 | 3.53E-36 | L1cam |
| ENSMUSG00000097068 | 506.6197386 | -7.686668537 | 6.67E-36 | Gm26760 |
| ENSMUSG00000011267 | 218.6846883 | 4.483095988 | 1.53E-35 | Zfp296 |
| ENSMUSG00000063234 | 308.5875141 | 5.535245542 | 3.22E-35 | Gpr84 |
| ENSMUSG00000021940 | 268.9375297 | -3.057618749 | 4.69E-35 | Ptpn20 |
| ENSMUSG00000000732 | 1733.43605 | 4.394241305 | 5.41E-35 | Icosl |
| ENSMUSG00000018965 | 36562.32048 | 2.136126696 | 2.94E-34 | Ywhah |
| ENSMUSG00000029819 | 5430.705627 | 9.003891375 | 2.94E-34 | Npy |
